# *Illicium verum* L. (Star Anise) Essential Oil: GC/MS Profile, Molecular Docking Study, In Silico ADME Profiling, Quorum Sensing, and Biofilm-Inhibiting Effect on Foodborne Bacteria

**DOI:** 10.3390/molecules28237691

**Published:** 2023-11-21

**Authors:** Emira Noumi, Iqrar Ahmad, Mohd Adnan, Harun Patel, Abderrahmen Merghni, Najla Haddaji, Nouha Bouali, Khulood Fahad Alabbosh, Adel Kadri, Lucia Caputo, Flavio Polito, Mejdi Snoussi, Vincenzo De Feo

**Affiliations:** 1 Department of Biology, College of Science, University of Hail, P.O. Box 2440, Ha’il 2440, Saudi Arabia; mo.adnan@uoh.edu.sa (M.A.); najla_haddaji@yahoo.fr (N.H.); nouhabouali82@gmail.com (N.B.); k.alabosh@uoh.edu.sa (K.F.A.); m.snoussi@uoh.edu.sa (M.S.); 2 Laboratory of Genetics, Biodiversity and Valorization of Bio-Resources (LR11ES41), Higher Institute of Biotechnology of Monastir, University of Monastir, Avenue Tahar Haddad, BP74, Monastir 5000, Tunisia; 3 Department of Pharmaceutical Chemistry, Prof. Ravindra Nikam College of Pharmacy, Gondur, Dhule 424002, Maharashtra, India; ansariiqrar50@gmail.com; 4 Division of Computer Aided Drug Design, Department of Pharmaceutical Chemistry, R. C. Patel Institute of Pharmaceutical Education and Research, Shirpur 425405, Maharashtra, India; hpatel_38@yahoo.com; 5 Laboratory of Antimicrobial Resistance LR99ES09, Faculty of Medicine of Tunis, University of Tunis El Manar, Tunis 1068, Tunisia; abderrahmen_merghni@yahoo.fr; 6 College of Science and Arts in Baljurashi, Al-Baha University, P.O. Box 1988, Al Baha 65527, Saudi Arabia; lukadel@yahoo.fr; 7 Department of Pharmacy, University of Salerno, Via Giovanni Paolo II, 132, 84084 Fisciano, Italy; lcaputo@unisa.it (L.C.); fpolito@unisa.it (F.P.)

**Keywords:** *Illicium verum*, chemical composition, pathogenic bacteria, antibiofilm, pharmacokinetics, in silico

## Abstract

*Illicium verum*, or star anise, has many uses ranging from culinary to religious. It has been used in the food industry since ancient times. The main purpose of this study was to determine the chemical composition, antibacterial, antibiofilm, and anti-quorum sensing activities of the essential oil (EO) obtained via hydro-distillation of the aerial parts of *Illicium verum*. Twenty-four components were identified representing 92.55% of the analyzed essential oil. (*E*)-anethole (83.68%), limonene (3.19%), and α-pinene (0.71%) were the main constituents of *I. verum* EO. The results show that the obtained EO was effective against eight bacterial strains to different degrees. Concerning the antibiofilm activity, trans-anethole was more effective against biofilm formation than the essential oil when tested using sub-inhibitory concentrations. The results of anti-swarming activity tested against *P*. *aeruginosa* PAO1 revealed that *I. verum* EO possesses more potent inhibitory effects on the swarming behavior of PAO1 when compared to trans-anethole, with the percentage reaching 38% at a concentration of 100 µg/mL. The ADME profiling of the identified phytocompounds confirmed their important pharmacokinetic and drug-likeness properties. The in silico study using a molecular docking approach revealed a high binding score between the identified compounds with known target enzymes involved in antibacterial and anti-quorum sensing (QS) activities. Overall, the obtained results suggest *I. verum* EO to be a potentially good antimicrobial agent to prevent food contamination with foodborne pathogenic bacteria.

## 1. Introduction

Antibiotic resistance presents a serious problem to human and environmental ecosystems [1]. The overuse of antibiotics to treat infectious diseases has contributed to the emergence of multidrug-resistant bacterial strains [2]. In recent years, the impact of antibiotics, which are used to inhibit microbial growth and slow fat oxidation in food, has been observed [3]. This has led the food industry to use natural resources such as essential oils in the preparation of foods [4]. This fact has generated a renewed interest in plant therapy medicine in recent decades [5]. The use of plants in alternative medicine has increased during the last 25 years [6]. Medicinal and aromatic plants (MAPs) are a rich reservoir of bioactive molecules which are able to promote health and can be used as drugs [7,8,9,10]. Essential oils and plant extracts represent an alternative to synthetic antioxidants and antimicrobial agents in the food industry as well as in the pharmaceutical industry, alternative medicine, and natural therapy.

Essential oils are a source of pharmaceutical materials required for the production of highly active antimicrobial agents based essentially on thymol, carvacrol, terpenoids, and eugenol. Furthermore, many researchers have investigated the modes of action of essential oils [11].

*Illicium verum*, also known as star anise, is the fruit of a medium-sized tree that grows in Asia and is native to China and Vietnam [12]. It is widely used as a culinary and medicinal fruit. The fruits are sweet and an important source of essential oil, with a wide range of culinary usages and medicinal properties such as carminative, digestive, antispasmodic, expectorant, antirheumatic, and diuretic properties. It relieves colic and is a common ingredient of cough lozenges and cattle sprays. Star anise oil has exhibited high antioxidant [13], insecticidal, fumigant [14], and antimicrobial activities [15].

Star anise seed oil is used worldwide as medicine [16]. The essential oil, rich in trans-anethole, is mainly used in the pharmaceutical and food industries [17]. The fruit contains tannins and essential oil (9–10%), consisting of anethole (85–90%), α-pinene, limonene, β-phellandrene, α-terpineol, and farnesol [18,19]. In a previous study, 16 compounds were identified in *I. verum* essential oil, among which trans-anethole was dominant (90.82%), followed by estragol (3.68%) [20].

Microorganisms are versatile pathogens that are transmitted through the soil, water, air, and food, and cause diseases in human beings and animals. It has been demonstrated that crude ethanol extract from the fruits of *I. verum* was effective against *S. aureus* ATCC 25923, *E. coli* ATCC 2592, and *P. aeruginosa* ATCC 27853. The antimicrobial activity of this fruit extract is due to the presence of anethole [21].

Microbial behavior and communication are regulated by the quorum-sensing (QS) systems, based essentially on the expression of virulence factors, including biofilm formation. In this context, the potential of star anise as an anti-QS and antibiofilm agent and its possible application in food safety can be investigated via the use of model bacteria *Pseudomonas aeruginosa* PAO1 and *Chromobacterium violaceum* [22].

Researchers are interested in the isolation and characterization of chemical constituents to study their biological activities. Nowadays, the molecular docking approach has become an important tool for drug discovery [23]. The phytochemical screening of plant compounds can provide information about biological effects of plant extracts and EO, but the phytoconstituents responsible for this action are still unknown [24]. Thus, in silico docking studies are essential to understand the affinities and the interactions between the identified compounds and target proteins [25,26,27].

The purpose of this work was to determine the active compounds in *I. verum* EO by using the GC-MS technique to further study its antibacterial and antibiofilm activities against several Gram-positive and -negative foodborne pathogenic bacteria. The ability of star anise EO and its main compounds to attenuate the quorum-sensing system was also tested using *Pseudomonas aeruginosa* and *Chromobacterium violaceum* strains. Moreover, the draggability and pharmacokinetic properties of *I. verum* EO were evaluated using ADME profiles and molecular docking approaches.

## 2. Results

### 2.1. Chemical Composition of I. verum EO

The chemical composition of *I. verum* EO is listed in Table 1. Twenty-four components were identified, representing 92.55% of the analyzed essential oil. *I. verum* EO was rich in (*E*)-anethole (83.68%), limonene (3.19%), and α-pinene (0.71%).

**Figure 1:**
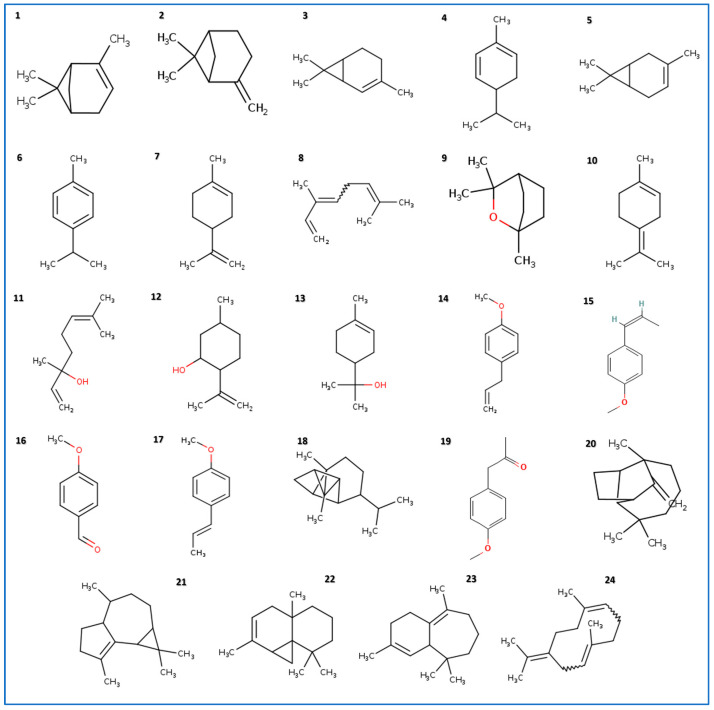
Chemical structure of the compounds identified in *I. verum* EO. All compounds’ names (**1**–**24**) are listed in Table 1.

All identified compounds in the EO of *I. verum* were divided into several chemical groups, including cyclic monoterpenes, such as limonene and α-pinene, and the monomethoxybenzene group, which is represented by the (*E*)-anethole. The chemical structures of all identified compounds are shown in Figure 1.

### 2.2. Antibacterial Potential of I. verum EO and Trans-Anethole

The antibacterial effect of *I. verum* EO and the trans-anethole was firstly assessed by means of the determination of inhibition zones (ZIs) on Mueller–Hinton (MH) agar. The obtained results of this test are summarized in Table 2. Except for *L. monocytogenes* CECT 933,120 and *S. aureus* ATCC 6538, trans-anethol EO with ZIs ranging from 6 ± 0.1 to 11.66 ± 0.57 mm showed less potent antibacterial effects compared to *I. verum* EO. The *I. verum* EO was found to be active only against three pathogenic strains (*L. monocytogenes*, *S. aureus*, and *S. enterica*), with ZIs > 9 mm. The MIC/MBC ratio showed that both tested agents exerted a bacteriostatic effect (MBC/MIC > 4) against all tested strains.

Using the microdilution technique (Table 2), the MICs of both *I. verum* EO and trans-anethole were about 0.048 mg/mL for bacterial strains and the MBCs were >50 mg/mL. Interestingly, a weak concentration of *I. verum* EO and trans-anethole (0.048 mg/mL) exhibited an inhibitory effect against the Gram-positive and Gram-negative pathogenic bacteria used in this study. Moreover, the tested essential oil showed bacteriostatic activity against almost all tested foodborne pathogenic bacteria, as the calculated values of MBC/MIC ratios were higher than four [28,29].

### 2.3. Adhesive Properties of Bacterial Strains

The results of slime production on Congo red agar (CRA) plates revealed that five (62.5%) strains were able to produce exopolysaccharides, displaying black or red-with-black-center colonies (Table 3).

Regarding the bacterial adhesiveness on polystyrene surfaces, evaluated using a CV staining assay, our results show that all strains are moderate biofilm producers (0.1 ≤ OD_570_ < 1) except for *S*. *aureus* ATCC 6538, which was found to be a highly active biofilm producer (OD_570_ ≥ 1) (Table 3).

**Figure 2:**
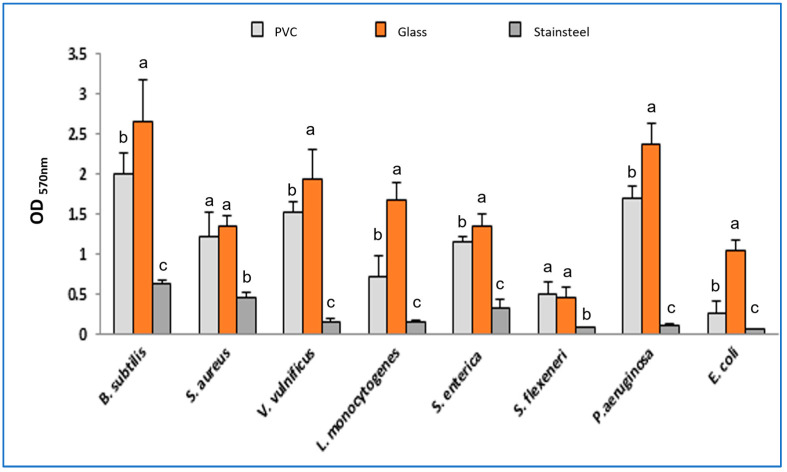
Biofilm formation ability of pathogenic bacteria on various materials. Values are the average of at least three independent determinations. Small letters are used to compare mean values of the optical density (OD_570 nm_) for each strain with three different materials. Means followed by the same letters are not significantly different at *p* < 0.05 based on Duncan’s multiple range test.

The biofilm formation on various abiotic surfaces revealed that most tested bacteria are able to produce biofilm structures on glass and polyvinyl chloride (PVC) materials. However, they showed low-grade biofilm formation on stainless steel surfaces (OD_570_ < 1). In addition, the tested Gram-positive bacteria showed more potent biofilm formation ability compared to the Gram-negative ones (Figure 2).

### 2.4. Antibiofilm Formation Activities

**Figure 3:**
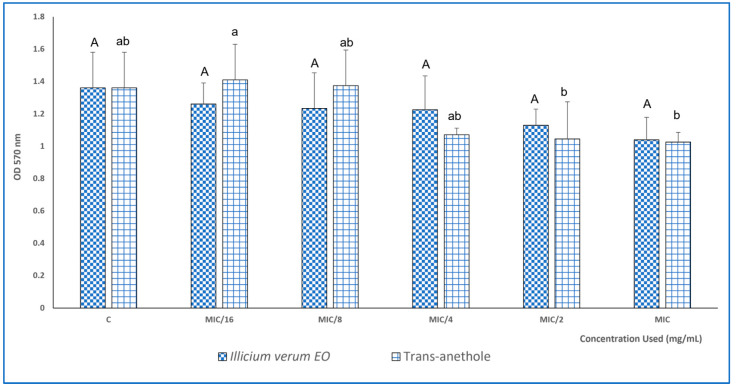
Biofilm inhibition capacity of *I. verum* EO and trans-anethole against *S. aureus* ATCC 6538. MIC = 0.048 mg/mL for the EO and trans-anethole. Values are the average of at least three independent determinations. Means followed by the same letters are not significantly different at *p* < 0.05 based on Duncan’s multiple range test. Small letters are used to compare mean values of different concentrations for trans-anethole, and capital letters are used to compare means between different concentrations of *I. verum* EO.

The capacity of *I. verum* EO and trans-anethole to inhibit biofilm formed by the *S. aureus* strain was also tested after treatment with different sub-inhibitory concentrations (1/16 × MIC to 1 × MIC). *S. aureus* ATCC 6538 biofilm was more affected by the tested EO, since at a concentration of MIC/16 (0.003 mg/mL), *I. verum* EO exhibited an antibiofilm effect in comparison with the untreated bacteria (control). The trans-anethole showed high biofilm inhibition starting from a concentration of about MIC/4 (0.012 mg/mL) to MIC (0.048 mg/mL) values (Figure 3).

**Figure 4:**
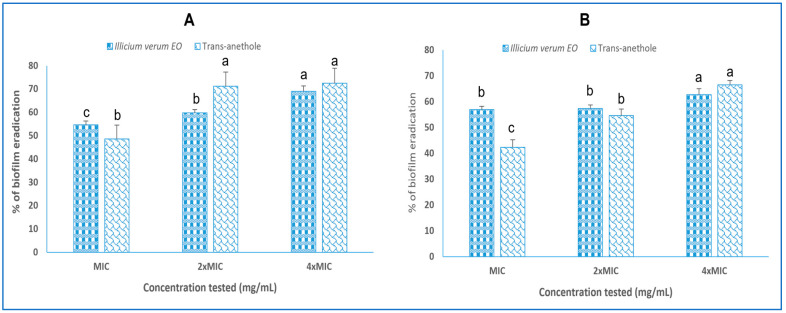
Biofilm eradication capacity of *I. verum* EO and trans-anethole on polystyrene (**A**) and glass (**B**) surfaces against *S. aureus* ATCC 6538. Values are the average of at least three independent determinations. Means followed by the same letters are not significantly different at *p* < 0.05 based on Duncan’s multiple range test. Small letters are used to compare mean values of different concentrations for trans-anethole and *I. verum* EO for each strain.

The ability to completely eradicate biofilm formed for 48 h by different concentrations of *I. verum* EO and trans-anethole ranging from MIC to 4 × MIC revealed that both tested agents showed high biofilm eradication on polystyrene and glass surfaces, with a percentage reduction exceeding 50% at a low concentration (1 × MIC) (Figure 4). Furthermore, at a high concentration (4 × MIC = 0.192 mg/mL), *I. verum* EO and trans-anethole showed similar effects against biofilm formation, with percentages of biofilm eradication reaching 70% (Figure 4).

### 2.5. Anti-Quorum Sensing Activities of the Tested Agents

#### 2.5.1. Anti-Swarming Activity

The results of anti-swarming activity tested against *P*. *aeruginosa* PAO1 revealed that trans-anethol induced more inhibitory effects on the swarming behavior of PAO1 when used at concentrations ranging between 50 µg/mL and 75 µg/mL. However, at a high concentration of 100 µg/mL, the *I. verum* EO showed a greater inhibition effect on the migration of PAO1, at 38 ± 0.9% (Table 4).

#### 2.5.2. Violacein Inhibition Assay

In qualitative analysis, MIC values of *I. verum* EO showed an inhibition of 76.18% in HSL-mediated violacein production. This inhibition was about 31.35% at MIC/2 of trans-anethole. A concentration of 0.3125 mg/mL (MIC/32) for the EO inhibited 3.67% of the bacterial growth (Table 5).

**Figure 5:**
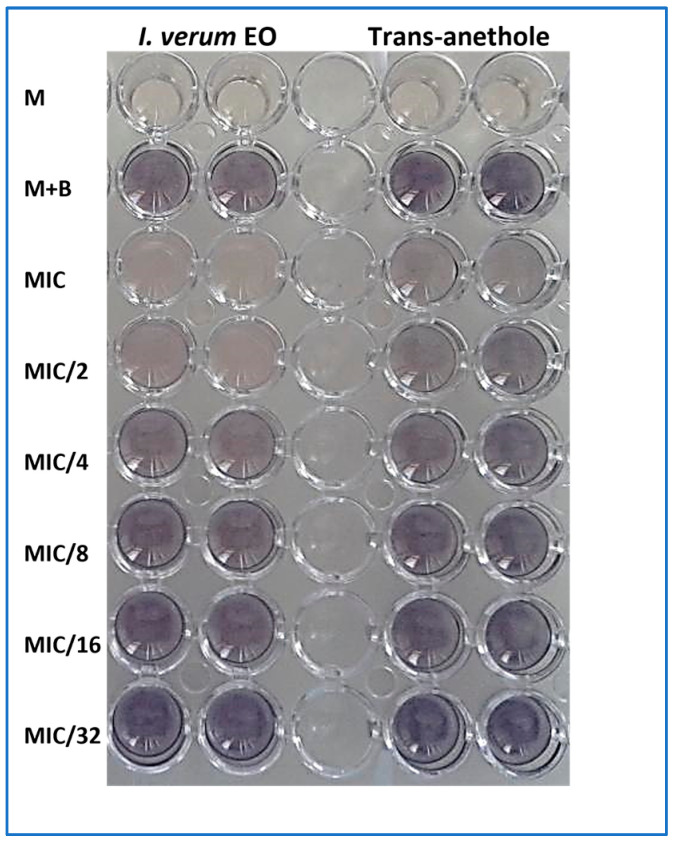
Effect of different MIC values of *I. verum* EO and trans-anethole on violacein inhibition in *C. violaceum* ATCC 12472.

The trans-anethole was able to inhibit violacein production by 11.67% at a concentration of about 0.156 mg/mL (MIC/8) (Table 5, Figure 5).

### 2.6. Druggability and Pharmacokinetic Properties of I. verum’s Main Compounds

**Figure 6:**
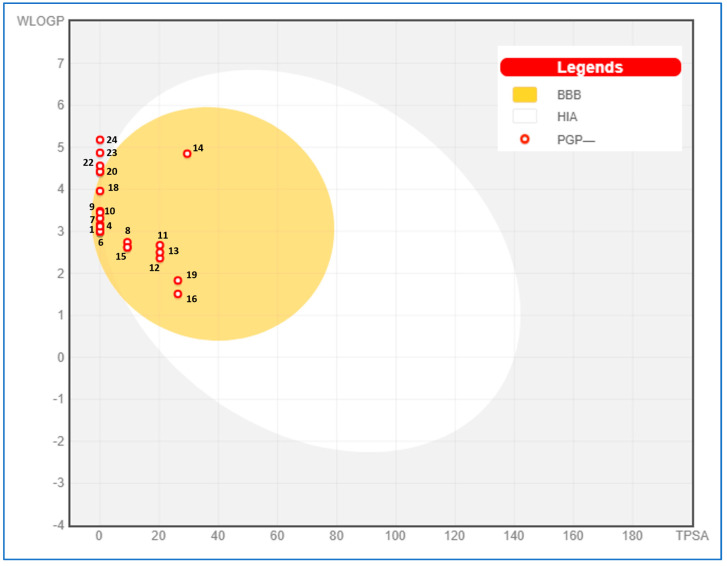
Boiled egg model of studied *I. verum* EO compounds. The names of the compounds are listed in Table 1.

The ADME properties of the twelve identified compounds were studied (Table 6). Compounds 20, 22, 23, and 24 (longifolene, cis-thujopsene, β-himachalene, and β-germacreme) were observed in the yellow zone of the boiled egg model (Figure 6). In addition, Lipinski’s rule was confirmed, and good gastro-intestinal absorption, lipophilicity, and bioavailability scores (0.55 to 0.58) were reported. In addition, all selected compounds exhibited good topological polar surface area values (TPSAs) lower than 125 Å^2^, suggesting that they are expected to be orally absorbed.

Similarly, all compounds were blood–brain barrier (BBB)-permeant. Interestingly, compounds 6 (ρ-cymene) and 14 (methyl chavicol) were able to inhibit four cytochrome P450 isoenzymes (CYP2D6). All selected compounds exhibited negative Log Kp values (skin permeability) ranging from −3.87 to −6.53, highlighting their suitability as good compounds to be delivered trans-dermally. All these results are summarized in Table 6.

**Figure 7:**
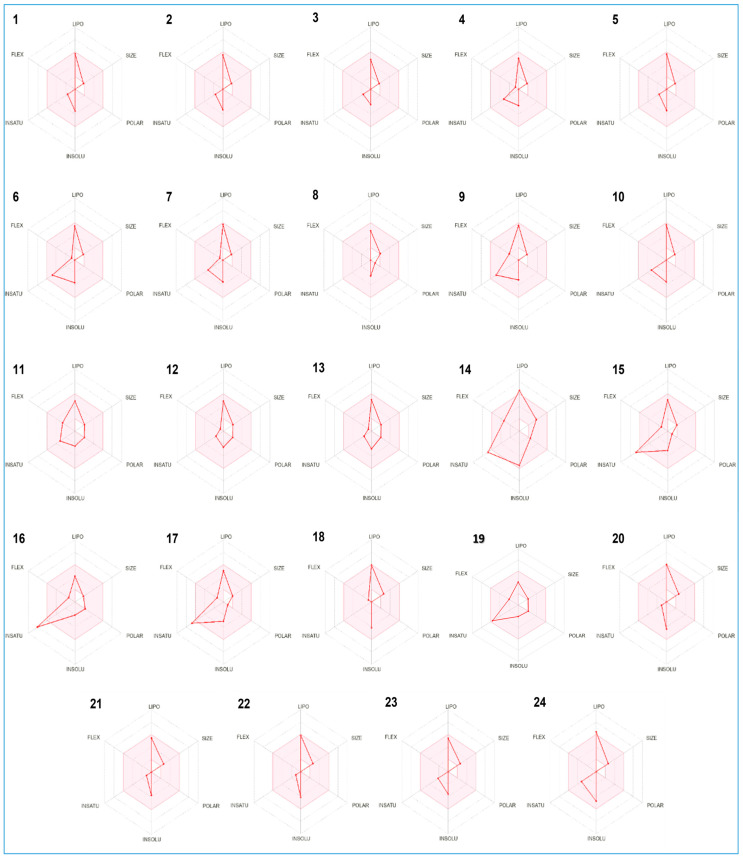
Bioavailability polygons of compounds identified in *I. verum* EO based on their physicochemical parameters using ADMET properties. Names of the compounds (**1**–**24**) are the same as in Table 1.

When analyzing the drug-likeness behavior of all identified compounds, represented by the bioavailability radar, we found that they all fit within the pink area of the bioavailability polygon (Figure 7).

### 2.7. Molecular Docking Study

A molecular docking study was conducted to determine the molecular binding affinities and optimal intermolecular interaction between the phytochemical ligand compounds and the target protein. The molecular docking study was carried out against five different receptors, namely the human peroxiredoxin 5 receptor (PDB: 1HD2), tyrosyl-tRNA synthetase (TyrRS) from *S. aureus* (PDB: 1JIJ), type IIA topoisomerase from *S. aureus* (PDB: 2XCT), and the LasR protein receptor of *P. aeruginosa* (PDB ID: 2UV0 and 3IX3). Table 7 summarizes the competitive analysis of the docking studies. As indicated in Table 7, all compounds displayed negative binding energies with the different target receptors (from −1.90 to −7.27 kcal/mol), with anisyl methyl ketone having the most promising docking score of all the receptors examined. On the other hand, α-pinene, β-pinene, δ-2-carene, α-phelandrene, δ-3-carene, and ρ-cymene showed significant docking scores on the 2XCT, 2UV0, and 3IX3 receptors, respectively, which are in the range of −5.51 to −6.97 kcal/mol. The binding affinity of phytochemicals in 1HD2 proteins varies from −3.03 to −5.105 kcal/mol, which is lower than the binding affinity of the target co-crystallized ligand, benzoic acid (−7.245 kcal/mol). Cyclosativene (−6.145 kcal/mol), anisyl methyl ketone (−7.215 kcal/mol), and β-himachalane (−6.429 kcal/mol) had the best docking scores in the 2XCT target.

**Figure 8:**
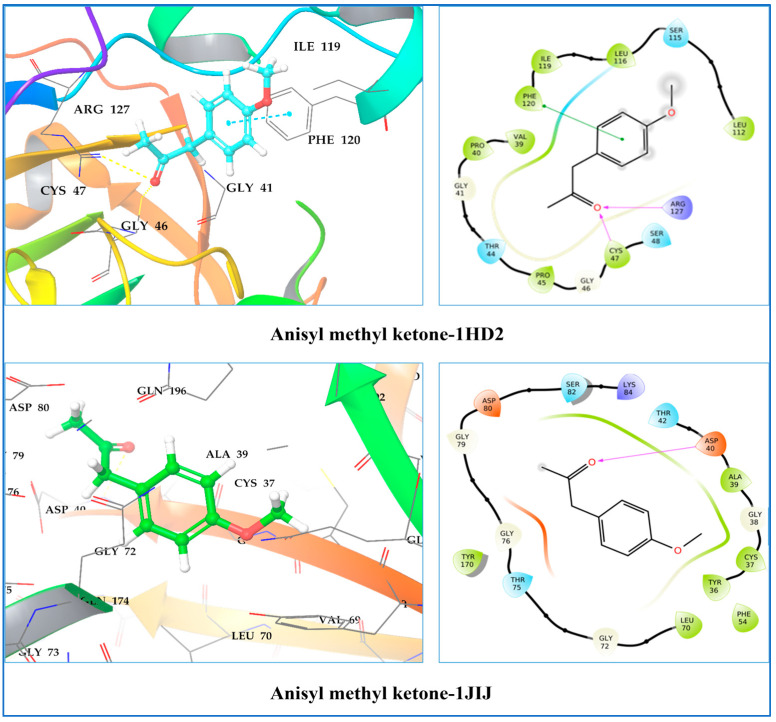
Two-dimensional and three-dimensional residual interaction network of the anisyl methyl ketone–1HD2 complex and anisyl methyl ketone–1JIJ complex.

The co-crystallized ligand benzoic acid had the highest docking score (−7.245 kcal/mol) in this target, whereas docking values ranging from −4.602 to −1.902 kcal/mol were revealed to have considerable binding affinities with anisyl methyl ketone (−4.308 kcal/mol) and α-phelandrene (−4.118 kcal/mol). Cys47, a cysteine residue common to all peroxiredoxins, has been connected to peroxide catalysis in the human peroxiredoxin family. At the N-terminus of the kinked helix 2 lies Cys47, a conserved cysteine residue. This active pocket is made up of conserved amino acid residues, including Thr44, Gly46, Cys47, and Arg127, which help with docked chemical identification through hydrogen bonding and hydrophobic interactions. Additionally, the complex benzoic acid–1HD2 stabilization involves Cys47, Thr44, Gly46, Thr147, Pro40, Pro45, Phe120, Arg127, and Leu149 (Figure 8). It was noticed that two typical hydrogen bonds developed in anisyl methyl ketone at Cys47 (2.22 Å) and Arg127 (2.62 Å). During the interaction, a Pi–Pi bond was also seen at the site of Met120 (4.32 Å). The presence of a hydrogen bond with Cys47 was also visible in the antioxidant reference ligand benzoic acid, indicating that these three phytoconstituents have antioxidant potential.

In *S. aureus*, TyrRS, the docking scores of phytoconstituents ranged from −5.644 to −1.927 kcal/mol, while the monocyclic SB-239629 co-crystallized ligand had a docking score of −7.973 kcal/mol. Through molecular docking analysis, it was found that α-pinene, β-pinene, δ-2-carene, α-phelandrene, δ-3-carene, ρ-cymene, terpinolene, ρ-anisaldehyde, cyclosativene, anisyl methyl ketone, α-gurjunene, cis-thujopsene, and β-himachalene were the compounds with the best binding affinity to the structures of TyrRS *S. aureus*. In this target, along with ρ-anisaldehyde, anisyl methyl ketone had the most significant docking score, which is −5.484 kcal/mol. The carbonyl group of anisyl methyl ketone formed a single hydrogen bond with the amino acid Asp40. Anisyl methyl ketone interacted with Thr42, Ala39, Gly38, Cys37, Tyr36, Leu70, Thr75, Gly76, Gly79, Asp80, Ser82, and Lys84 at the binding site via van der Waals bonding (Figure 8).

**Figure 9:**
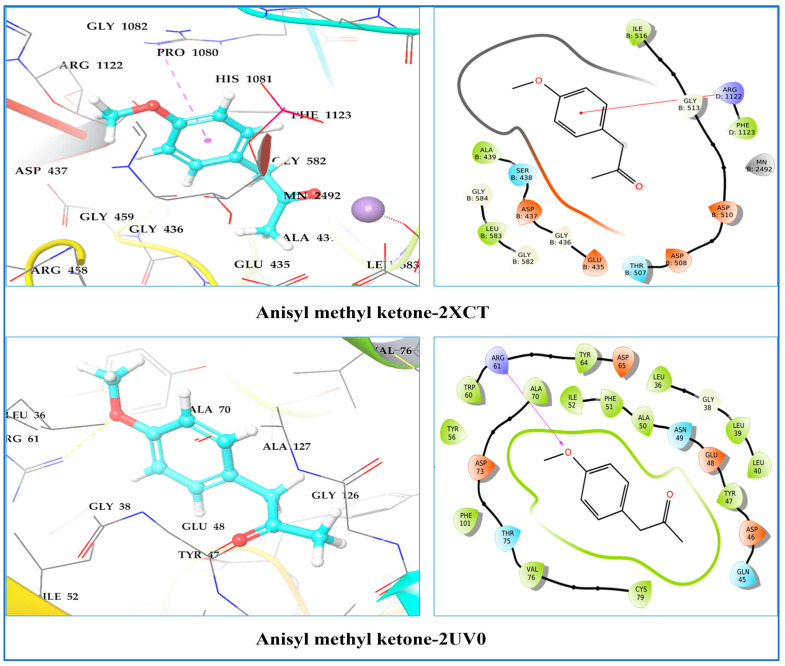
Two-dimensional and three-dimensional residual interaction network of the anisyl methyl ketone–2XCT complex and anisyl methyl ketone–2UV0 complex.

DNA topoisomerases address topological problems that occur during processes such as DNA replication, transcription, recombination, chromatin assembly, and chromosomal segregation. Consequently, this enzyme is essential for bacterial survival and could be employed predominantly as an antibacterial targeted treatment. The highest scoring ligands in the docking study on topoisomerase II DNA gyrase (2XCT) protein were anisyl methyl ketone (−7.215 kcal/mol), α-terpineol (−6.551 kcal/mol), isopulegol (neoiso) (−6.512 kcal/mol), β-himachalene (−6.429 kcal/mol), α-phelandrene (−6.372 kcal/mol), terpinolene (−6.241 kcal/mol), ρ-cymene (−6.204 kcal/mol), δ-2-carene (−6.193 kcal/mol), δ-3-carene (−6.193 kcal/mol), and cyclosativene 6.145 kcal/mol). The binding interaction of the most active ligand, anisyl methyl ketone, revealed that there was no direct hydrogen bonding between the ligand atom and protein residues, although Arg1112 exhibit π–cation interaction (Figure 9).

Quorum sensing is a process of cell–cell communication that allows bacteria to share information about cell density and adjust gene expression accordingly. Since *P. aeruginosa* is an invasive human illness that kills thousands of cystic fibrosis patients and other immunocompromised individuals each year, its quorum-sensing system is presently the most intensively investigated. The virulence of *P. aeruginosa* may be lowered by utilizing quorum-sensing inhibitors. Docking experiments on the *P. aeruginosa* LasR protein receptor (PDB ID: 2UV0 and 3IX3) were carried out to evaluate quorum-sensing inhibition activity. Anisyl methyl ketone was chosen as the best natural ligand against 2UV0 and 3IX3 because of its good binding affinities within the active site domains, which were −7.277 kcal/mol and −7.303 kcal/mol, respectively, comparable to a co-crystallized ligand. In the *P. aeruginosa* 2UV0 protein target, anisyl methyl ketone formed one hydrogen bond with Arg61 and van der Waals contacts with Ser40, Gln45, Asp46, Tyr47, Glu48, Asn49, Ala50, Phe51, Ile52, Val76, Ser77, Cys79, and Thr80, which is a similar binding site to that of a co-crystallized ligand (Figure 9).

**Figure 10:**
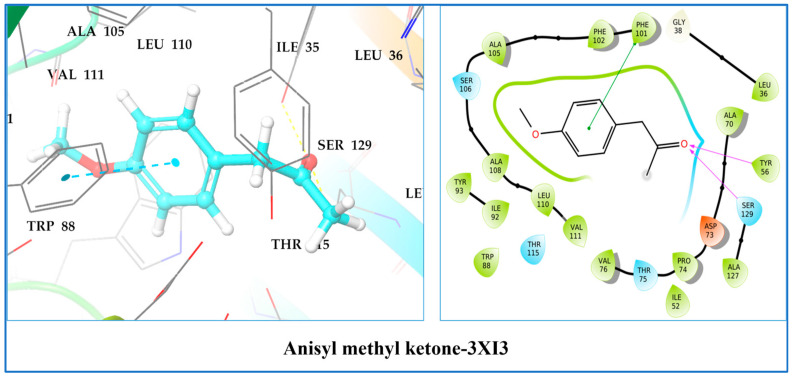
Two-dimensional and three-dimensional residual interaction network of anisyl methyl ketone–3IX3.

One perpendicular π–π contact was also seen in this complex with the hydrophobic residue Phe101 (Figure 10).

The presence of anisyl methyl ketone in the hydrophobic pocket of the LasR receptor suggests the presence of hydrophobic contacts formed by the benzofuran scaffold and hydrophobic amino acids. As a result, molecular docking studies have shown that the structure of the phytocompound anisyl methyl ketone has the potential to suppress the quorum-sensing mechanism.

## 3. Discussion

In this work, we used the GC/MS technique to identify the active compounds in *I. verum* EO. The major compounds were (*E*)-anethole (83.68%), limonene (3.19%), and α-pinene (0.71%). Several studies have shown similar results to our study and identified the same composition in star anise EO, with percentages of trans-anethole ranging from 88.5% to 92.4%, which is known for its important biological activities, namely antimicrobial and antioxidant [30,31,32]. Soher et al. [33] demonstrated that star anise EO was rich in trans-anethole (82.7%), carryophyllene (4.8%), and limonene (2.3%). The dominant component of the EO obtained from *I. verum* fruit is the phenylpropanoid compound trans-anethole. The average content of trans-anethole in the *I. verum* EO is around 72–92% [34]. The variations in the amounts of chemical compounds of *I. verum* EO can be explained by the climatic conditions, cultivation period, extraction method, and storage process of the EO [35,36]. *I. verum* is an important medicinal plant characterized by diverse biological activities such as antibacterial, antifungal, anti-inflammatory, and antioxidant properties [37]. This plant is also used as a spice in the food industry [38]. In the present study, we confirmed the antibacterial activity of star anise EO. Freire et al. [39] demonstrated that the EO of *I. verum* was effective against *Escherichia coli* strains. In fact, it has been observed that the sensitivity of Gram-positive bacteria is higher than that of Gram-negative bacteria [40,41]. This was the case in the present study—in fact, *Staphylococcus aureus* (Gram-positive bacteria) was more sensitive to the action of *I. verum* EO compared to Gram-negative bacteria (*Pseudomonas aeruginosa* PAO1, *Shigella flexeneri*, and *Vibrio vulnificus*). Ebani et al. [42] demonstrated that the EO from *I. verum* exhibited antibacterial activity against many bacterial strains, except for *Enterococcus* [42]. The *I. verum* aqueous methanol extract possesses antibacterial activity against multidrug-resistant *Acinetobacter baumannii* and methicillin-resistant *Staphylococcus aureus* [43]. It has been reported that the antimicrobial activity of star anise is essentially due to anethole [44].

Gene expression is regulated by the mechanism of bacterial cell communication. It has been demonstrated that the signal molecules of bacteria and the suppression of QS can be affected by some plants’ compounds [45]. The interaction between plants and bacteria has been studied for many years [45]. During our study, we investigated the anti-QS potential of *I. verum* EO and trans-anethole in vitro. This plant is known for its culinary uses as a spice and flavoring, and as a medicinal plant. The results show that *I. verum* EO and trans-anethole inhibited violacein production in *C. violaceum* ATCC 12,472, highlighting its ability to inhibit bacterial cell-to-cell communication, and consequently interfering with the QS system regulating the production of virulence traits responsible for bacterial disease [46]. Computational studies are commonly used to correlate between the in vitro activities of natural compounds with key target proteins involved in human disorders. In fact, in silico docking studies can provide useful insights into the molecular basis of the biological activity of natural products and the possible mechanisms of action and binding modes of active compounds. Therefore, all compounds from the GC-MS analysis of compounds from the tested essential oil were docked with specific target proteins involved in antibacterial and antioxidant activities [47,48]. In 2017, Rahman et al. reported that *I. verum* extracts may serve as potential quorum-sensing and biofilm inhibitors [22].

Phytochemical screening identifies a plant extract’s biological activity but does not determine which phytoconstituent is active. As a consequence, understanding the interaction and affinity of identified phytoconstituents with biological targets requires in silico docking investigations. An in silico docking analysis may provide valuable insights into the molecular basis of natural product biological activity, as well as putative mechanisms of action and binding modalities of active compounds. As a result, all compounds identified in the GC-MS analysis of the ethanolic extract were docked to determine the binding affinity and potential interaction between the identified phytoconstituents and certain target proteins implicated in antibacterial and antioxidant activities.

Human peroxiredoxin 5 (1HD2), distributed mostly in the mitochondria, peroxisomes, and cytosol, is associated with antioxidant protection processes as well as signal transmission in cells, making it an appropriate target for measuring antioxidant activity [49].

It was noticed that two typical hydrogen bonds developed in anisyl methyl ketone at Cys47 (2.22 Å) and Arg127 (2.62 Å). During the interaction, a Pi–Pi bond was also seen at the site of Met120 (4.32 Å). The presence of a hydrogen bond with Cys47 was also visible with the antioxidant reference ligand benzoic acid, indicating that these three phytoconstituents have antioxidant potential.

Antimicrobial medications often obstruct cell wall production, protein synthesis, nucleic acid synthesis, and anti-metabolism. In general, antibiotics disrupt these processes by interfering with particular cell proteins that perform specific functions. Tyrosyl-tRNA synthetase (TyrRS), an aminoacyl-tRNA synthetase family member, can interpret information such as contemporaneous tRNA molecules and amino acid structures, both of which are required for converting coded information into protein structures in nucleic acids [50]. Since this enzyme is highly conserved across prokaryotes, it is a potential target for the creation of broad-spectrum antibiotics. Phyto-constituents from an ethanolic extract were docked in the active site of the crystal structure of *S. aureus* TyrRS (1JIJ), which was co-crystallized with the monocyclic SB-239629, to understand better the binding interactions [51].

Anisyl methyl ketone is located on the two DNA helices and interacts with nucleotide bases through van der Waals interactions. Anisyl methyl ketone binding contact was unable to engage with the Mn + 2 ion through a salt bridge, resulting in much higher rates of enzyme-mediated DNA breaking than previously reported [31,52].

In this target co-crystalized ligand, acylhomoserine lactone is housed in a large hydrophobic pocket formed by residues Leu36, Gly38, Leu39, Leu40, Tyr47, Glu48, Ala50, Ile52, Tyr56, Trp60, Arg61, Tyr64, Asp65, Gly68, Tyr69, Ala70, Asp73, Pro74, Thr75, Val76, Cys79, Thr80, Trp88, Tyr93, Phe101, Phe102, Ala105, Leu110, Thr115, Leu125, Gly126, Ala127, and Ser129. Meanwhile, in 3IX3 protein, anisyl methyl ketone is situated in a cage of hydrophobic and acidic amino acids, like Tyr69, Ala70, Asp73, Thr75, Val76, Ser44, Gln45, Asp46, Tyr47, Glu48, Asn49, Ala50, Phe51, and Ile52 [53].

## 4. Materials and Methods

### 4.1. Chemical Composition Analyses

*Illicium verum* EO and its main compound trans-anethole were purchased from Huile & Sens (Crestet, France) and Sigma (Sigma-Aldrich S.r.l. Milan, Italy), respectively. The chemical composition of the essential oil was analyzed using a gas chromatography–flame ionization detector (GC–FID) and GC–MS [54].

### 4.2. Disk Diffusion Test

The antagonistic effects of *I. verum* EO and trans-anethole were evaluated against eight pathogenic bacterial strains: *Listeria monocytogenes* CECT 933; *Vibrio vulnificus* CECT 529; *Salmonella enterica* CECT 443; *Shigella flexeneri* CECT 4804; *Staphylococcus aureus* ATCC 6538; *Bacillus subtilis* CIP 5265, *Escherichia coli* ATCC 35,218, and *Pseudomonas aeruginosa* PAO1 [55]. All reference bacterial strains were obtained from the American Type Culture Collection (ATCC) and Spanish Type Culture Collection (CECT). The obtained bacterial strains were maintained on Muller–Hinton agar (MHA) plates. Gentamicin discs were used as positive controls.

### 4.3. Microdilution Assay

The minimal inhibition concentration (MIC) and the minimal bactericidal concentration (MBC) of *I. verum* EO and its main compounds were determined for all bacterial strains as previously described [56]. Serial dilution of the tested agents was performed in concentrations ranging from 50 to 0.048 mg/mL.

### 4.4. Adhesive Potentiality

The ability of the eight tested microorganisms to secrete exopolysaccharides on Congo red agar plates was determined, and the morphotypes obtained were defined on the basis of their color (slime production) using the protocol described by Touati et al. [57]. Qualitative adhesion on glass tubes was carried out following the same protocol described in [57]. All experiments were performed in triplicate.

Quantitative biofilm formation on polystyrene 96-well plates was determined using the crystal violet technique [58].

### 4.5. Biofilm Formation Capacity on Abiotic Surfaces

Polyvinyl chloride (PVC), stainless steel (SS), and glass (G) strips (1.5 cm^2^) were disinfected before being used for the biofilm assay. Bacterial suspensions with a volume of 100 µL were added onto each strip placed on a 12-well tissue culture plate. After incubation (24 h at 37 °C), non-adherent cells were removed from each well by washing with PBS solution. Biofilm quantification was performed with crystal violet (1%) staining and then dissolved into acetic acid (33%). The OD at 570 nm was recorded [56].

### 4.6. Antibiofilm Activities

#### 4.6.1. Biofilm Inhibition

The biofilm inhibition effects of *I. verum* EO and trans-anethole were evaluated against *S. aureus* ATCC 6538 based on its ability to highly produce biofilm on polystyrene [58]. The selected strain grown in BHI (with 2% glucose) was treated with different subinhibitory concentrations (1/16 to 1 × MIC) of the tested agents. After incubation for 24 h at 37 °C, non-adherent cells were removed, and CV (1%)-stained biofilm cells were determined at 570 nm.

#### 4.6.2. Biofilm Eradication

The biofilm eradication properties of *I. verum* EO and trans-anethole were tested against *S. aureus* ATCC 6538 as described previously [59]. Pre-established biofilms (48 h) were treated with various concentrations ranging from MIC to 4 × MIC of the selected agents and further incubated for 24 h. The treated biofilm biomass was stained with CV (1%) and measured according to the absorbance of CV at 570 nm. The percentage of biofilm eradication was estimated as:

[(OD growth control − OD sample)/OD growth control] × 100.(1)

### 4.7. Anti-Quorum Sensing Activity

The ability of *I. verum* EO and trans-anethole to inhibit the production of the water-soluble pigment (violacein) in the *Chromobacterium violaceum* ATCC 12,472 starter strain was evaluated. An overnight culture of *C. violaceum* (OD_600_ = 0.4) was added into sterile microtiter plates containing 200 μL of LB broth and incubated at 30 °C supplemented with different concentrations (MIC/32 to MIC) of star anise EO and trans-anethole. LB broth containing *C. violaceum* was used as a positive control [58]. The percentage of violacein reduction was calculated using the following formula:

Violacein inhibition (%) = (OD_585_ nm Control − OD_585_ nm Sample)/OD_585_ nm Control.

### 4.8. Anti-Swarming Activity

The anti-swarming motility assay of *I. verum* EO and trans-anethole was assessed against *Pseudomonas aeruginosa* PAO1. Five microliters of an overnight culture at 30 °C of PAO1 (OD600 = 0.4) was point-inoculated at the center of swarming plates consisting of 1% peptone, 0.5% NaCl, 0.5% agar, and 0.5% filter-sterilized D-glucose at various concentrations of test agents (50, 75, and 100 μg/mL). Plates were incubated at an appropriate temperature in an upright position for 18 h. The swarming migration was recorded by following swarm fronts of the bacterial cells. Plates without EOs were considered as controls [60].

### 4.9. ADMET Profile

The pharmacokinetics and the toxicity profiles of the identified molecules were predicted using a SwissADME online server (http://www.swissadme.ch/, accessed on 19 January 2022) and pkCSM webserver (https://biosig.lab.uq.edu.au/pkcsm/, accessed on 19 January 2022) [60,61,62].

### 4.10. Molecular Docking Study

To highlight the possibility of binding interactions between the phytocompounds identified in *I. verum* EO and antimicrobial activity, antioxidant activity, and QS receptors, a docking approach was utilized. For antimicrobial activity, *S. aureus* tyrosyl-tRNA synthetase (PDB ID, 1JIJ) and topoisomerase II DNA gyrase (PDB ID, 2XCT) proteins are promising drug candidates leading to high selectivity and a broad spectrum of antibacterial agents [63,64]. Human peroxiredoxin 5 (PRDX5) receptor (PDB ID, 1HD2) is a potential target for the evaluation of antioxidant activity of selected bioactive compounds which permits the reduction of hydrogen peroxide and alkyl peroxide, with the help of thiol-containing donor molecules [64,65]. A molecular docking study was also performed against the QS signal receptor LasR (PDB IDs: 2UV0 and 3IX3) as a key regulator of *P. aeruginosa* pathogenesis.

The phytoconstituents’ 2D structures were obtained using PubChem chemical information resources. The LigPrep module was used to refine the obtained structure. The OPLS3e force field was applied for ligand preparation. Tautomer creation, ionization state at pH 7.0 ± 1.0 utilizing Epik, charged group neutralization, and optimization of the hydrogen bond and ligand 3D geometry were all included in the ligand preparation process [66,67]. Proteins with PDB IDs 1HD2, 1JIJ, 2XCT 2UV0, and 3IX3 were downloaded from the Protein Data Bank and processed for the modeling study. 1HD2 includes peroxidases able to reduce hydrogen peroxide and alkyl hydroperoxides with the goal of reducing equivalents derived from thiol-containing donor molecules. 1JIJ is considered as a class of potent and specific inhibitors of bacterial tyrosyl-tRNA synthetases. Proteins belonging to 2XCT are able to cleave and relegate DNA to regulate DNA topology and are a major class of antibacterial and anticancer drug targets. 2UV0 and 3IX3 are QS signal receptors of LasR in *P. aeruginosa*, regulating its pathogenesis. The proteins were imported into the Schrodinger Maestro GUI and refined, optimized, and minimized after undesired water molecules and problem warnings were removed using the Protein Preparation Wizard module [64,65]. The Prime tool was applied to complete the missing side chains and residues. The OPLS3e force field was utilized to construct low-energy state proteins with a default root mean square deviation (RMSD) of 0.30 Å, which were then employed for molecular docking.

A grid was constructed from minimized proteins, and the grid box was constructed based on the active site of the protein where the co-crystallized ligand was bound [68]. Glide was used to undertake molecular docking simulations using the standard precision (SP) approach, which also produced favorable ligand poses for further evaluating active sites for ligand binding. The docking results included the best positions, as well as the dock score.

### 4.11. Statistical Analysis

All experiments were performed in triplicate, and average values were calculated using the SPSS 25.0 statistical package for Windows. Differences in means were calculated using Duncan’s multiple range tests for means with a 95% confidence interval (*p* ≤ 0.05). For anticancer activities, a significance test was carried out among the treatments using two-way ANOVA followed by Bonferroni’s post hoc test at *p* < 0.001.

## 5. Conclusions

*Illicium verum* is a plant which is very rich in phytochemicals that are characterized by a large spectrum of biological activities. Docking studies on the identified phytocompounds in bacteria were carried out to reinforce the in vitro results. The obtained results confirm the alternative of use of this plant to treat human diseases because of its effectiveness and safety. *I. verum* EO is a considerable natural antibacterial agent and might be used as a natural preservative in the food industry.

## Figures and Tables

**Table 1:** Chemical composition of *I. verum* EO using the GC–EIMS technique.

No.	Compound	Percentage	Ki ^a^	Ki ^b^
1	α-pinene	0.71	924	939
2	β-pinene	0.10	983	979
3	δ-2-carene	0.30	994	1002
4	α-Phelandrene	0.46	1000	1002
5	δ-3-carene	0.15	1007	1011
6	ρ-cymene	0.12	1015	1024
7	Limonene	3.19	1020	1029
8	1,8-cineole	0.34	1022	1031
9	(*E*)-β-ocimene	0.10	1050	1050
10	Terpinolene	0.13	1080	1088
11	Linalool	0.29	1093	1096
12	Isopulegol (neoiso)	0.22	1170	1171
13	α-terpineol	0.28	1183	1188
14	Methyl chavicol	0.37	1191	1196
15	*Z*-anethole	0.21	1246	1252
16	ρ-anisaldehyde	0.33	1250	1250
17	(*E*)-anethole	83.68	1288	1284
18	Cyclosativene	0.11	1369	1371
19	Anisyl methyl ketone	0.15	1378	1382
20	Longifolene	0.10	1408	107
21	α-gurjunene	0.20	1412	1409
22	*cis*-thujopsene	0.23	1429	1431
23	β-himachalene	0.14	1501	1501
24	β-germacreme	0.08	1556	1561

^a^ Kovats retention index determined relative to the retention time (*t_R_*) of a series of n-alkanes (C10–C35) on an HP-5 MS column; ^b^ Kovats retention index determined relative to the *t_R_* of a series of n-alkanes (C10–C35) on HP Innowax.

**Table 2:** Antibacterial activity of *I. verum* EO and trans-anethole against pathogenic bacteria recorded as inhibition zones.

Strains	*I. verum* EO			MBC/MIC Ratio	Trans-Anethole			MBC/MIC Ratio
	IZ ± SD (mm)	MIC (mg/mL)	MBC (mg/mL)		IZ ± SD (mm)	MIC (mg/mL)	MBC (mg/mL)	
*Listeria monocytogenes* CECT 933	9.33 ± 0.57	0.048	>50	>4; Bacteriostatic	11.66 ± 0.57	0.048	50	>4; Bacteriostatic
*Vibrio vulnificus* CECT 529	8 ± 0.1	0.048	>50	>4; Bacteriostatic	6 ± 0.1	0.048	>50	>4; Bacteriostatic
*Shigella flexeneri* CECT 4804	8 ± 0.1	0.097	>50	>4; Bacteriostatic	6 ± 0.1	0.048	>50	>4; Bacteriostatic
*Bacillus subtilis* CIP 5265	10 ± 0.1	0.048	>50	>4; Bacteriostatic	6 ± 0.1	0.048	50	>4; Bacteriostatic
*Salmonella enterica* CECT 443	10.33 ± 0.57	0.048	>50	>4; Bacteriostatic	6 ± 0.1	0.048	>50	>4; Bacteriostatic
*Escherichia coli* ATCC 35218	12 ± 1	0.048	>50	>4; Bacteriostatic	9 ± 0.1	0.048	50	>4; Bacteriostatic
*Pseudomonas aeruginosa* PAO1	7.33 ± 0.57	0.048	25	>4; Bacteriostatic	6 ± 0.1	0.048	>50	>4; Bacteriostatic
*Staphylococcus aureus* ATCC 6538	13.66 ± 0.57	0.048	>50	>4; Bacteriostatic	8.33 ± 0.57	0.048	>50	>4; Bacteriostatic

MIC and MBC values. IZ: inhibition zone; SD: standard deviation; MIC: minimum inhibitory concentration; MBC: minimum bactericidal concentration.

**Table 3:** Adhesive properties of selected pathogenic strains.

Strains	Slime Production on CRA		Adhesion to Polystyrene	
	Color	S+/S−	OD_570_ ± SD	Biofilm Production
*S. aureus* ATCC 6538	Black	S+	1.36 ± 0.2	High producer
*L. monocytogenes* CECT 933	Red with black center	S+	0.19 ± 0.07	Moderate producer
*V. vulnificus* CECT 529	Red with black center	S+	0.13 ± 0.02	Moderate producer
*B. subtilis* CIP 5265	Bordeaux red	S−	0.12 ± 0.01	Moderate producer
*E. coli* ATCC 35218	Red with black center	S+	0.17 ± 0.03	Moderate producer
*S. flexeneri* CECT 4804	Red with black center	S+	0.10 ± 0.01	Moderate producer
*S. enterica* CECT 443	Bordeaux red	S−	0.15 ± 0.01	Moderate producer
*P. aeruginosa* PAO1	Bordeaux red	S−	0.42 ± 0.26	Moderate producer

**Table 4:** Effect of *I. verum* EO and trans-anethole on swarming motility of PAO1.

Tested Agent	Concentrations	% Anti-Swarming Activity
	50 µg/mL	15.5 ± 1.3
***Illicium verum***	75 µg/mL	19.7 ± 2.6
	100 µg/mL	38 ± 0.9
	50 µg/mL	20.83 ± 4.17
**Trans-anethole**	75 µg/mL	25.61 ± 1.2
	100 µg/mL	33.33 ± 0

**Table 5:** Qualitative violacein inhibition on *C. violaceum* ATCC 12472. MIC: minimum inhibitory concentration; MIC *I. verum* EO: 10 mg/mL; MIC trans-anethole: 1.25 mg/mL.

Concentration	% of Violacein Inhibition	
	*I. verum* EO	Trans-Anethole
MIC	76.18 ± 1.1	48.78 ± 1.1
MIC/2	67.61 ± 1	31.35 ± 1.1
MIC/4	46.77 ± 1.3	19.12 ± 1.3
MIC/8	23.6 ± 1	11.67 ± 1
MIC/16	17.33 ± 1.2	8.87 ± 1.2
MIC/32	3.67 ± 1.4	1.1 ± 0.67

**Table 6:** Selected ADME properties of identified compounds in *I. verum* EO. The number and name of the compounds are the same as listed in Table 1.

Entry	1	2	3	4	5	6	7	8	9	10	11	12
**Physicochemical Properties**												
Molecular weight (g/mol)	136.23	136.23	136.23	136.23	136.23	134.22	136.23	154.25	136.23	136.23	154.25	154.25
Num. heavy atoms	10	10	10	10	10	10	10	11	10	10	11	11
Num. arom. heavy atoms	0	0	0	0	0	6	0	0	0	0	0	0
Fraction Csp3	0.80	0.80	0.80	0.60	0.80	0.40	0.60	1.00	0.40	0.60	0.60	0.80
Num. rotatable bonds	0	0	0	1	0	1	1	0	3	0	4	1
Num. H-bond acceptors	0	0	0	0	0	0	0	1	0	0	1	1
Num. H-bond donors	0	0	0	0	0	0	0	0	0	0	1	1
Molar refractivity	45.22	45.22	45.22	47.12	45.22	45.99	47.12	47.12	48.76	47.12	50.44	48.76
TPSA (Å^2^)	0	0	0	0	0	0	0	9.23	0	0	20.23	20.23
Consensus log Po/w	3.44	3.42	3.12	2.97	3.42	3.5	3.37	2.67	3.4	3.4	2.66	2.42
Lipinski rules	Yes	Yes	Yes	Yes	Yes	Yes	Yes	Yes	Yes	Yes	Yes	Yes
Bioavailability score	0.55	0.55	0.55	0.55	0.55	0.55	0.55	0.55	0.55	0.55	0.55	0.55
**Pharmacokinetics**												
GI absorption	Low	Low	Low	Low	Low	Low	Low	High	Low	Low	High	High
BBB permeant	Yes	Yes	Yes	Yes	Yes	Yes	Yes	Yes	Yes	Yes	Yes	Yes
P-gp substrate	No	No	No	No	No	No	No	No	No	No	No	No
CYP1A2 inhibitor	No	No	No	No	No	No	No	No	No	No	No	No
CYP2C19 inhibitor	No	No	No	No	No	No	No	No	No	No	No	No
CYP2C9 inhibitor	Yes	Yes	No	No	Yes	No	Yes	No	No	Yes	No	No
CYP2D6 inhibitor	No	No	No	No	No	Yes	No	No	No	No	No	No
CYP3A4 inhibitor	No	No	No	No	No	No	No	No	No	No	No	No
Log K_p_ (cm/s)	−3.95	−4.18	−5.11	−4.85	−4.02	−4.21	−3.89	−5.30	−4.11	−3.96	−5.13	−5.15
Entry	13	14	15	16	17	18	19	20	21	22	23	24
**Physicochemical Properties**												
Molecular weight (g/mol)	154.25	296.4	148.2	136.15	148.2	204.35	164.20	204.35	204.35	204.35	204.35	204.35
Num. heavy atoms	11	22	11	10	11	15	12	15	15	15	15	15
Num. arom. heavy atoms	0	12	6	6	6	0	6	0	0	0	0	0
Fraction Csp3	0.80	0.20	0.20	0.12	0.20	1.00	0.30	0.87	0.87	0.87	0.73	0.60
Num. rotatable bonds	1	5	2	2	2	1	3	0	0	0	0	0
Num. H-bond acceptors	1	2	1	2	1	0	2	0	0	0	0	0
Num. H-bond donors	1	1	0	0	0	0	0	0	0	0	0	0
Molar refractivity	48.80	94.58	47.83	38.32	47.83	65.24	47.71	66.88	67.14	66.62	68.78	70.68
TPSA (Å^2^)	20.23	29.46	9.23	26.3	9.23	0	26.30	0	0	0	0	0
Consensus log Po/w	2.58	4.53	2.79	1.61	2.79	4.32	1.94	4.5	4.27	4.48	4.20	4.60
Lipinski rules	Yes	Yes	Yes	Yes	Yes	Yes	Yes	Yes	Yes	Yes	Yes	Yes
Bioavailability score	0.55	0.55	0.55	0.55	0.55	0.55	0.55	0.55	0.55	0.55	0.55	0.55
**Pharmacokinetics**												
GI absorption	High	High	High	High	High	Low	High	Low	Low	Low	Low	Low
BBB permeant	Yes	Yes	Yes	Yes	Yes	Yes	Yes	No	No	No	No	No
P-gp substrate	No	No	No	No	No	No	No	No	No	No	No	No
CYP1A2 inhibitor	No	Yes	Yes	Yes	Yes	Yes	Yes	No	No	No	No	No
CYP2C19 inhibitor	No	Yes	No	No	No	Yes	No	Yes	Yes	Yes	No	No
CYP2C9 inhibitor	No	Yes	No	No	No	Yes	No	Yes	Yes	Yes	Yes	Yes
CYP2D6 inhibitor	No	Yes	No	No	No	No	No	No	No	No	No	No
CYP3A4 inhibitor	No	No	No	No	No	No	No	No	No	No	No	No
Log K_p_ (cm/s)	−4.83	−3.87	−4.86	−5.88	−4.86	−4.09	−6.16	−3.94	−4.64	−4.17	−4.75	−3.45

**Table 7:** Docking score (kcal/mol) of identified phytoconstituents with the antioxidant, antibacterial, and anti-QS activity targets.

Sr. No.	Compound	1HD2	1JIJ	2XCT	2UV0	3IX3
1	α-pinene	−3.86	−4.526	−5.707	−6.614	−6.585
2	β-pinene	−3.597	−4.499	−5.512	−6.574	−6.546
3	δ-2-carene	−3.86	−4.526	−6.193	−6.614	−6.585
4	α-phelandrene	−4.118	−4.788	−6.372	−6.688	−6.69
5	δ-3-carene	−3.814	−4.591	−6.193	−6.97	−6.75
6	ρ-cymene	−3.336	−4.481	−6.204	−6.727	−6.774
7	Limonene	−3.032	−3.521	−5.525	−6.153	−5.874
8	1,8-cineole	−3.696	−4.724	−5.026	−6.355	−5.949
9	(*E*)-β-ocimene	−1.902	−1.927	−3.782	−3.89	−2.913
10	Terpinolene	−3.551	−5.061	−6.241	−6.695	−6.592
11	Linalool	−2.829	−3.051	−4.889	−4.752	−3.896
12	Isopulegol (neoiso)	−3.385	−4.508	−6.512	−6.47	−6.223
13	α-terpineol	−4.46	−4.811	−6.551	−5.536	−6.092
14	Methyl chavicol	−4.02	−4.575	−5.947	−6.147	−5.861
15	*Z*-anethole	−3.444	−4.022	−5.89	−5.916	−5.999
16	ρ-anisaldehyde	−4.602	−5.644	−5.573	−5.724	−5.635
17	(*E*)-anethole	−3.037	−3.973	−5.921	−4.504	−5.529
18	Cyclosativene	−3.512	−5.147	−6.145	−6.204	−6.237
19	Anisyl methyl ketone	−4.308	−5.484	−7.215	−7.277	−7.303
20	Longifolene	−3.698	−3.686	−5.396	−6.657	−5.623
21	α-gurjunene	−3.871	−4.436	−4.321	−5.232	−6.542
22	*cis*-thujopsene	−3.639	−4.264	−5.472	−5.727	−5.662
23	β-himachalene	−3.673	−5.073	−6.429	−6.303	−6.834
24	*β*-germacreme	−3.536	−3.566	−5.908	−6.715	−5.915
-	Co-crystal inhibitor	−7.245	−7.973	−8.521	−6.929	−6.057

## Data Availability

Data is contained within the article.
